# Supplementary material for: Parallel G-quadruplexes recruit the HSV-1 transcription factor ICP4 to promote viral transcription in herpes virus-infected human cells
Source: Commun Biol. 2021 Apr 30;4:510. doi: 10.1038/s42003-021-02035-y (PMC8087788; doi:10.1038/s42003-021-02035-y)
Supplement: Supplementary file 1 — Supplementary Information [file 42003_2021_2035_MOESM1_ESM.pdf]

Supporting Information

Parallel G-quadruplexes recruit the HSV-1 transcription factor ICP4 to promote viral transcription in herpes virus-infected human cells

Ilaria Frasson, Paola Soldà, Matteo Nadai, Sara Lago, Sara N. Richter*

Department of Molecular Medicine, University of Padua, via A. Gabelli 63, 35121 Padua, Italy

Table S1. Oligonucleotides used in this study

Assay	Name	Sequence (5'-3')	260/290	T_m (°C)
CD	un2	GGGGGCGAGGGGCGGGAGGGGGCGAGGGG	-0.33	>90
	un2L2	GGGGGCGAGGGGCGGGAGGGGGCGAGGGGGGGGGCGAGGGGCGGGAGGGGGCGAGGGG	11.20	>90
	myc	GGGGAGGGTGGGGAGGGTGGGGAAGG	18.66	>90
	LTR-III	GGGAGGCGTGGCCTGGGCGGGACTGGGG	0.78	65.5 ± 0.1
	hTel	GGGTTAGGGTTAGGGTTAGGG	0.24	68 ± 0.1
	ICP4-146532	GGGCGGGGCGCGAGGGCGGGTGGG	3.20	79.5 ± 0.1
	ICP4-146666	GGGGTGGGCCCGCCGGGGGGGCGGGGGG	8.71	>90
	ICP4-146574-8	GGGCGGGGCCGGGGGTTCGACCAACGGGCCGCGGCCACGGG	2.09	76.02 ± 1.53
	ICP4-146947	GGCGGGGGTCGTCGGGGTCCGTGGG	5.30	69.2 ± 0.2
FRET/ EMSA	F-un2-T	FAM-GGGGGCGAGGGGCGGGAGGGGGCGAGGGG-TAMRA	25.04	74.41 ± 5.10
	F-myc-T	FAM-GGGGAGGGTGGGGAGGGTGGGGAAGG-TAMRA	52.19	78.8 ± 12.45
	F-LTR-III-T	FAM-GGGAGGCGTGGCCTGGGCGGGACTGGGG-TAMRA	1.25	65.80 ± 1.23
	F-hTel-T	FAM-GGGTTAGGGTTAGGGTTAGGG-TAMRA	0.26	67.38 ± 1.05
	un2 complementary	CCCCTCGCCCCCTCCCGCCCCTCGCCCCC	nd	nd
	myc complementary	CCTTCCCCACCCTCCCCACCCTCCCC	nd	nd
	LTR-III complementary	CCCCAGTCCCGCCCAGGCCACGCCTCCC	nd	nd
	hTel complementary	CCCTAACCCTAACCCTAACCC	nd	nd
	F-un2	FAM-GGGGGCGAGGGGCGGGAGGGGGCGAGGGG	nd	nd
	F-G-rich-scrambled	FAM-GGAGTCGTGTCGCGTGTCGAGCGTGTGTAGTGG	nd	nd
Pull-down/MS XL pull-down/CD	B-un2	Btn-TTTTTGGGGGCGAGGGGCGGGAGGGGGCGAGGGG	8.32	>90
	B-un2L2	Btn-TTTTTGGGGGCGAGGGGCGGGAGGGGGCGAGGGGGGGGGCGAGGGGCGGGAGGGGGCGAGGGG	10.92	>90
	B-myc	Btn-TTTTTGGGGAGGGTGGGGAGGGTGGGGAAGG	13.67	>90
	B-gp054dL3	Btn-TTTTTGGGGTTGGGGCTGGGGTTGGGGGGGGTTGGGGCTGGGGTTGGGGGGGGTTGGGGCTGGGGTTGGGG	3.12	>90
	B-LTR-III	Btn-GGGAGGCGTGGCCTGGGCGGGACTGGGG	0.84	68.80 ± 0.77
	B-LTR-II+III+IV	Btn-TTTTTGGGGACTTTCCAGGGAGGCGTGGCCTGGGCGGGACTGGGGAGTGG	1.39	63.07 ± 0.79
	B-ICP4-146532	BtnTg-GGGCGGGGCGCGAGGGCGGGTGGG	59.56	>90
	B-ICP4-146666	BtnTg-GGGGTGGGCCCGCCGGGGGGGCGGGGGG	73.11	>90
	B-ICP4-146574-8	BtnTg-GGGCGGGGCCGGGGGTTCGACCAACGGGCCGCGGCCACGGG	11.44	72.16 ± 1.18
	B-ICP4-146947	BtnTg-GGCGGGGGTCGTCGGGGTCCGTGGG	10.43	>90
	B-IE3	Btn-TTTTTCCGATCGTCCACACGGAGC	0.42	nd
	IE3 complementary	GCTCCGTGTGGACGATCGGAAAAA		
	B-G-rich-scrambled	Btn-TTTTTGGAGTCGTGTCGCGTGTCGAGCGTGTGTAGTGGTTTTT	-0.03	nd
	B-BOM17	Btn-GGTTAGGTTAGGTTAGG	-0.32	40.16 ± 0.45
	B-VEGF	Btn-CGGGGCGGGCCGGGGGCGGGG	14.56	>90
	B-LTR-IIIc	Btn-CCCCAGTCCCGCCCAGGCCACGCCTCCC	0.19	72.37 ± 8.46

	B-gp054a	Btn-GGGGTTGGGGCTGGGGTTGGGG	0.22	88.1 ± 1.3
FISH; PLA	B-un2 shifted-PLA	Btn Tg-GTTTATTTCGAGGGGCGG	nd	nd

T_m: melting temperatures (°C), calculated from at least two independent CD thermal unfolding experiments. Nd = not detected. Btn: Biotin, BtnTg: Biotin TEG, FAM: 6-carboxyfluorescein, TAMRA: 6-carboxy-tetramethylrhodamine

Table S2. Proteins recovered in the pull-down/MS analysis with the four G4 baits

G4 bait	Origin	Protein acronym	Gene name	Protein match	Score/Match 8 h.p.i. Elution 1 G4	8 h.p.i. Elution 1 G-rich	8 h.p.i. Elution 2 G4	8 h.p.i. Elution 2 G-rich	16 h.p.i. Elution 1 G4	16 h.p.i. Elution 1 G-rich	16 h.p.i. Elution 2 G4	16 h.p.i. Elution 2 G-rich
un2L2	Viral	PAP	UL42	DNA polymerase processivity factor	212/16	ND	280/17	69/13	331/10	ND	191/9	160/6
	Viral	ICP4	ICP4	Infected Cell Polypeptide 4	186/25	ND	235/18	ND	196/11	ND	97/5	ND
	Viral	MCP	UL19	Major capsid protein	ND	ND	ND	ND	277/14	67	240/13	ND
	Viral	dUTPase	DUT	Deoxyuridine 5'-triphosphate nucleotidohydrolase	99/13	ND	32/5	ND	ND	ND	ND	ND
	Viral	TK	UL23	Thymidine kinase	96/7	ND	ND	ND	82/2	ND	ND	ND
	Cellular	hnRNP A1L2	HNRNPA1 L2	Heterogeneous nuclear ribonucleoprotein A1 like 2	ND	ND	41/9	ND	ND	ND	ND	ND
	Viral	TRX-1	UL38	Triplex capsid protein-1	ND	ND	ND	ND	ND	ND	59/2	ND
	Viral	TRX-2	UL18	Triplex capsid protein-2	ND	ND	ND	ND	ND	ND	36/4	ND
	Cellular	C23	NCL	Nucleolin	31/9	ND	ND	ND	151/6	114/4	ND	ND
gp054dL3	Viral	ICP4	ICP4	Infected Cell Polypeptide 4	87/4	ND	36/2	ND				
	Viral	PAP	UL42	DNA polymerase processivity factor	64/4	ND	ND	ND				
	Viral	MCP, VP5	UL19	Major capsid protein	ND	ND	35/2	ND				
LTR-III	Viral	ICP4	ICP4	Infected Cell Polypeptide 4	45/3	ND	ND	ND				
myc	Viral	TK	UL23	Thymidine kinase	112/3	ND	ND	ND				
	Viral	ICP4	ICP4	Infected Cell Polypeptide 4	97/5	ND	ND	ND				
	Viral	PAP	UL42	DNA polymerase processivity factor	75/6	ND	42/3	48/3				
	Viral	MCP, VP5	UL19	Major capsid protein	ND	ND	72/4	ND				

Protein matches were obtained in two independent experiments. The indicated oligonucleotides were used as G4 baits, while a G-rich oligonucleotide unable to fold into G4 (G-rich scrambled in Table S1) was used as control. Protein hits with scores lower than 30 were not retained, nor those displaying scores higher than 30 in the interaction with the magnetic streptavidin-coated matrix. The two displayed numbers were assigned by Mascot software: Score indicates the probability that the observed match is not a random event; Match indicates the number of fragments that match the recognized protein. ND not detected.

Table S3. FRET analysis

Oligonucleotides and proteins	E	ΔE	R (Å)	% unfolding (ΔE)	% unfolding (R)
**un2**	**0.66±0.003**	**0.30±0.017**	**44.66±0.122**	**0**	**0**
ds un2	0.36±0.020	0	55.14±0.801	100	100
un2 + ipICP4 (5x)	0.62±0.022	0.04±0.002	45.94±0.071	13.33	16.6
un2 + ipICP4 (10x)	0.58±0.008	0.08±0.012	47.40±0.261	26.66	26.14
un2 + ufICP4 (10x)	0.32±0.003	0.34±0.001	56.60±0.132	114.28	102.64
un2 + ipICP4 (5x) + complementary strand	0.61±0.001	0.05±0.018	46.18±0.055	16.66	14.50
un2 + ipICP4 (10x) + complementary strand	0.52±0.001	0.14±0.018	49.02±0.015	46.66	41.60
un2 + ufICP4 (10x) + complementary strand	0.31±0.002	0.35±0.001	56.70±0.115	113.33	102.80
un2 + BSA (10x)	0.66±0.005	0	44.61±0.128	0	0.47
un2 + complementary strand	0.63±0.090	0.03±0.070	45.54±0.081	10	8.39
un2 + BSA + complementary strand	0.64±0.011	0.02±0.008	45.29±0.036	6.66	5.7
un2 + B19	1.01±0.007	-0.35±0.004	37.51±0.229	-53.03	- 16
un2 + ufICP4 (10x) + B19	0.40±0.008	0.16±0.005	47.41±0.283	47.05	26.24
**myc**	**0.60±0.008**	**0.22±0.008**	**46.63±0.004**	**0**	**0**
ds myc	0.38±0.009	0	54.55±0.180	100	100
myc + ipICP4 (10x)	0.18±0.003	0.42±0.006	63.80±0.238	190	116.95
myc + ufICP4 (10x)	0.12±0.002	0.48±0.007	68.96±0.260	218	126.4
myc + ipICP4 (10x) + complementary strand	0.13±0.019	0.47±0.010	68.29±0.198	213	125.1
myc + pICP4 (10x) + complementary	0.11±0.001	0.49±0.008	70.32±0.002	222	128.9
myc + BSA (10x)	0.62±0.001	0.02±0.007	45.89±0.017	9	9.81
myc + complementary strand	0.57±0.006	0.03±0.003	47.51±0.210	13.6	11.67
myc + BSA + complementary strand	0.57±0.003	0.03±0.006	47.61±0.099	13.6	12.99
**LTR-III**	**0.83±0.005**	**0.82±0.004**	**38.34±0.026**	**0**	**0**
ds LTR-III	0.01±0.001	0	102.56±0.298	100	100
LTR-III + ipICP4 (10x)	0.86±0.004	0.03±0.001	39.27±0.175	3.6	2.4
LTR-III + ufICP4 (10x)	0.82±0.003	0.01±0.002	38.47±0.178	1.21	0.2
**hTel**	**0.82±0.003**	**0.81±0.001**	**38.52±0.150**	**0**	**0**
hTel + ufICP4 (10x)	080±0.002	0.02±0.002	39.36±0.924	2.1	2.4
ds hTel	0.004±0.002	0	126.53±0.937	100	100
hTel + ipICP4 (10x)	0.79±0.003	0.03±0.001	39.73±0.163	3.6	3.1
hTel + BSA (r=10)	0.82±0.003	0	38.60±0.145	0.2	0
hTel + pICP4 (10x)	080±0.002	0.02±0.001	39.36±0.924	2.1	2.4

Energy Transfer (E), Energy Transfer Difference (ΔE) and Radius (Å) of G4-folding oligonucleotides from two independent experiments were calculated in the absence/presence of ICP4/BSA proteins and complementary strand. The percentage of unfolding was calculated on the ΔE and R values of the ds oligonucleotide. ipICP4 = immunopurified ICP4; ufICP4 = ultrafiltrated ICP4; BSA = Bovine Serum Albumin; ds = double-stranded.

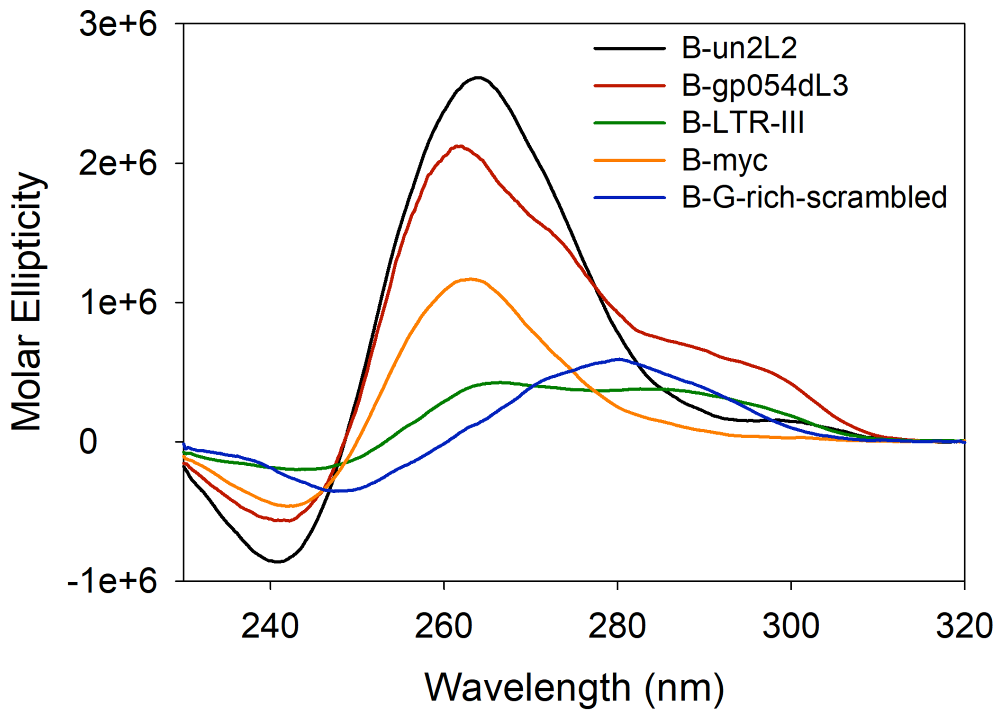

Figure S1. CD spectra of the biotinylated oligonucleotides used in the pull-down/MS assay. Oligonucleotides were folded into G4 in potassium phosphate buffer (20 mM PB, 80 mM KCl). Oligonucleotide folding was tested in two independent assays, one replicate per condition. The figure shows spectra of one measurement per oligonucleotide.

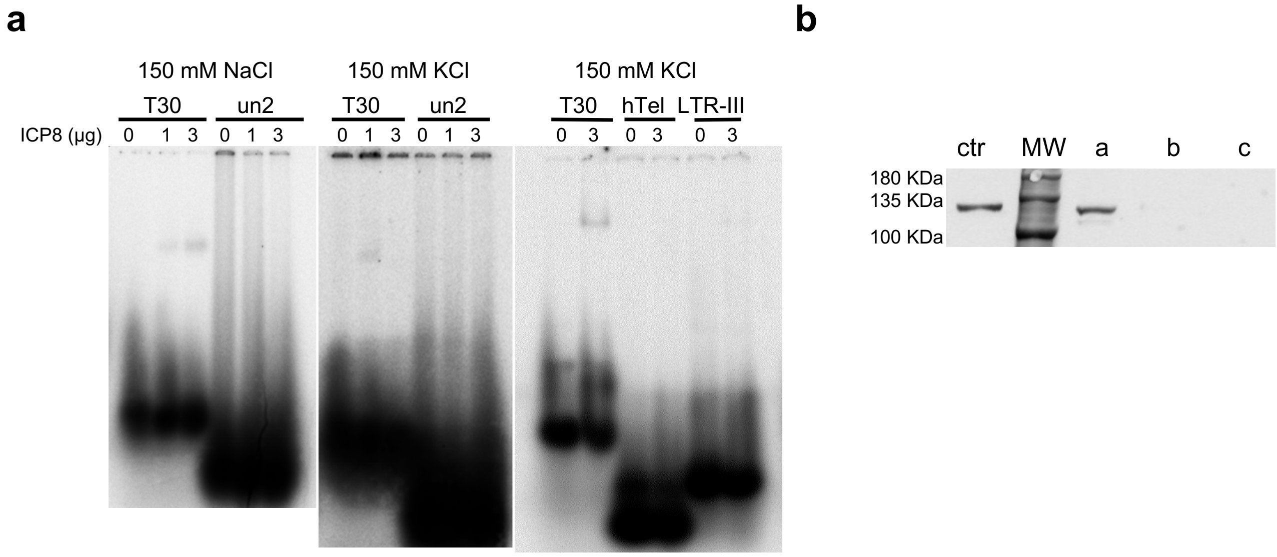

Figure S2. **ICP8 does not bind the G4 structures**. (**a)** EMSA of ^{33}P-labelled PolyT (T30) and HSV-1 un2, HIV-1 LTR-III and hTel G4 after 30 min incubation with increasing amounts of ICP8 (0-3 µg), in 20 mM Tris–HCl, pH 8, 30 mM KCl, 1.5 mM MgCl2, 1 mM ZnOAc, 8% glycerol, 1% Phosphatase Inhibitor Coktail I, 5 nM NaF, 1mM Na3VO4, 2.5 ng/ml poly dI-dC. The analyses were carried out in 8% polyacrylamide gel (29:1) in TBE (150 mM NaCl or 150 mM KCl, as indicated) 1x at 4°C. G4 oligonucleotides were folded overnight in 100 mM KCl. EMSA assays were performed in two independent experiments, with one replicate per condition. Images from one experiment are shown. **(b)** Western blot analysis of the pull-down assay of un2 G4 in presence of infected cells nuclear extracts (NE, 10 µg), ctr: control (NE 10 µg), lane a: unbound fraction; lane b. washing with 20 mM PB 150 mM NaCl; lane c. elution in sample buffer (50 mM Tris-HCl pH 6.8, 1% SDS, 6% glycerol, 0.1% DTT, 12.5 mM EDTA, 0.02 % bromophenol blue); MW: molecular weight Marker VI (Applichem). Results were acquired from two independent experiments, with one replicate per condition. The Western-blot analysis from one experiment is shown.

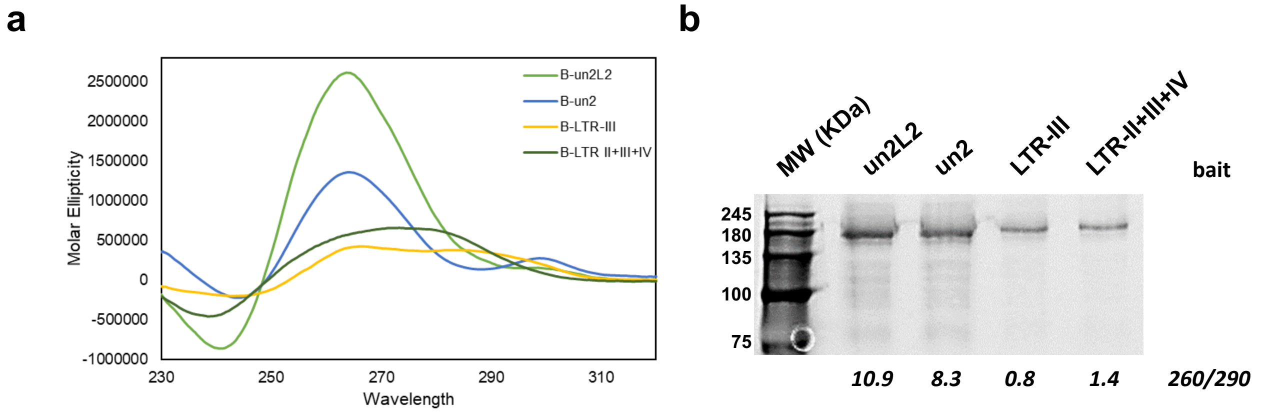

Figure S3. ICP4 binding to G4s is independent of oligonucleotide length. (a) CD spectra of the biotinylated oligonucleotides used in the pull-down assay. Oligonucleotides were folded into G4 in potassium phosphate buffer (20 mM PB, 80 mM KCl). Oligonucleotide folding was tested in two independent assays, one replicate per condition. The figure shows spectra of one measurement per oligonucleotide. **(b)** Western Blot analysis of ICP4 binding to the indicated G4s that have the same core G4 sequence and different length (un2L2 = 58 nts, un2 = 29 nts, LTR-III = 28 nts, LTR-II+III+IV = 45 nts). Infected cell nuclear extracts (5 µg) were incubated with formaldehyde-activated G4-folded oligonucleotides; proteins bound to the G4s were eluted after high stringency washes. ICP4 was detected by the anti-ICP4 antibody. MW: molecular weight (Marker VI, Applichem). Below the gel image, the 260/290 ratio for each biotinylated oligonucleotide is displayed. Results were acquired from two independent experiments, with one replicate per condition. Western-blot analysis from one experiment is shown.

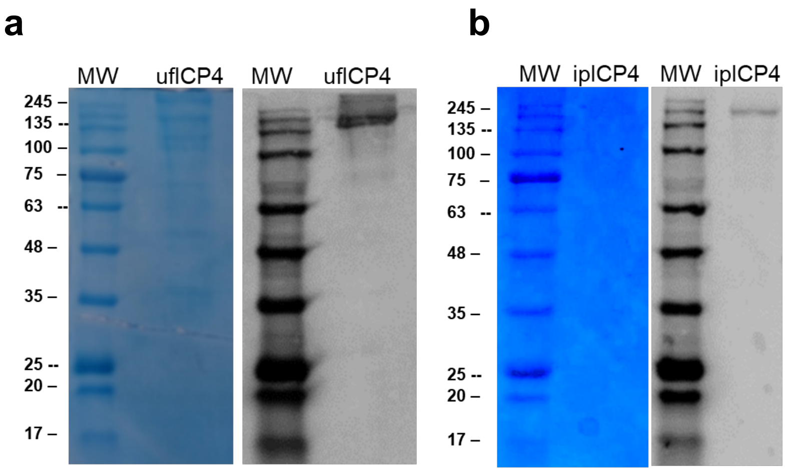

c

Protein	Mass (Da)	Match	Score
Filamin-A OS=Homo sapiens	283301	2/2	35
Major viral transcription factor ICP4 OS=Human herpesvirus 1 (strain 17)	133618	13/13	136
Transitional endoplasmic reticulum ATPase OS=Homo sapiens	89950	1/1	31

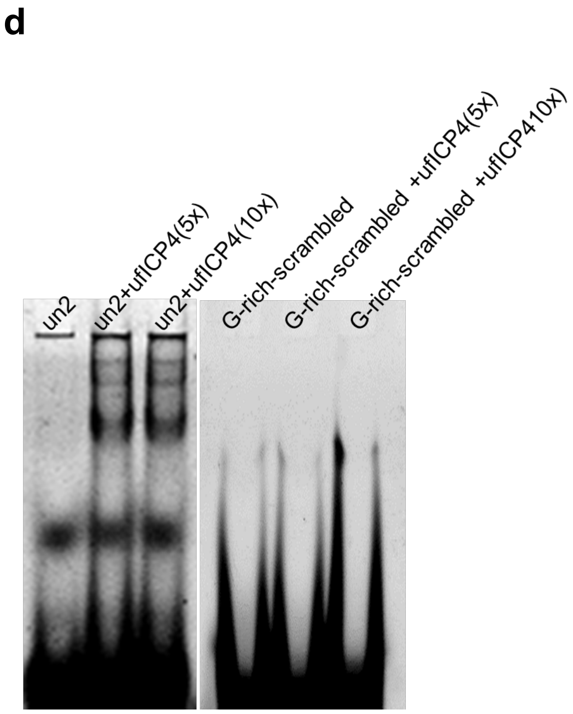

Figure S4. ICP4 purification from infected cells. Comassie stained SDS-PAGE and Western blot of the ultrafiltrated ICP4 (ufICP4, panel **a**) and immunoprecipitated ICP4 (ipICP4, panel **b**) using a monoclonal anti-ICP4 antibody. MW, molecular weight marker IV (Applichem). Each purification was performed starting from 20 µg of total nuclear extracts (NE) of infected U-2 OS cells (HSV-1 MOI 2, 6hpi). Each purified ICP4 aliquot was run on 10% SDS-PAGE gel. The polyacrylamide gel was subsequently divided into two parts: one was fixed in 25% MetOH and 10% HOAC and stained in Comassie solution (10% acetic acid in water, containing

60 mg/L of Coomassie Blue R-250), whereas the second part was transferred to a nitrocellulose membrane and analysed via WB. UfICP4 was also subjected to whole bands digestion and LC-MS/MS analysis MW is the molecular weight marker (Applichem, Protein Marker VI (10 – 245 KDa. Both purifications procedures were performed in more than three independent experiments with one replicate. Gels from one experiment are shown. **(c)** MS analysis of the ultrafiltered protein fraction obtained in panel **b**. **(d)** EMSA of F-un2 and F-G-rich-scrambled after 45 min incubation with increasing amounts of ufICP4 (5-10X). The analyses were carried out in 5% polyacrylamide gel (29:1) in phosphate buffer (50 mM PB, pH 7.4) at 4°C. G4 oligonucleotides were folded overnight in 20 mM PB, pH 7.4 and 80 mM KCl. The assay was performed in two independent experiments with one replicate per condition. Gels from one experiment are shown.

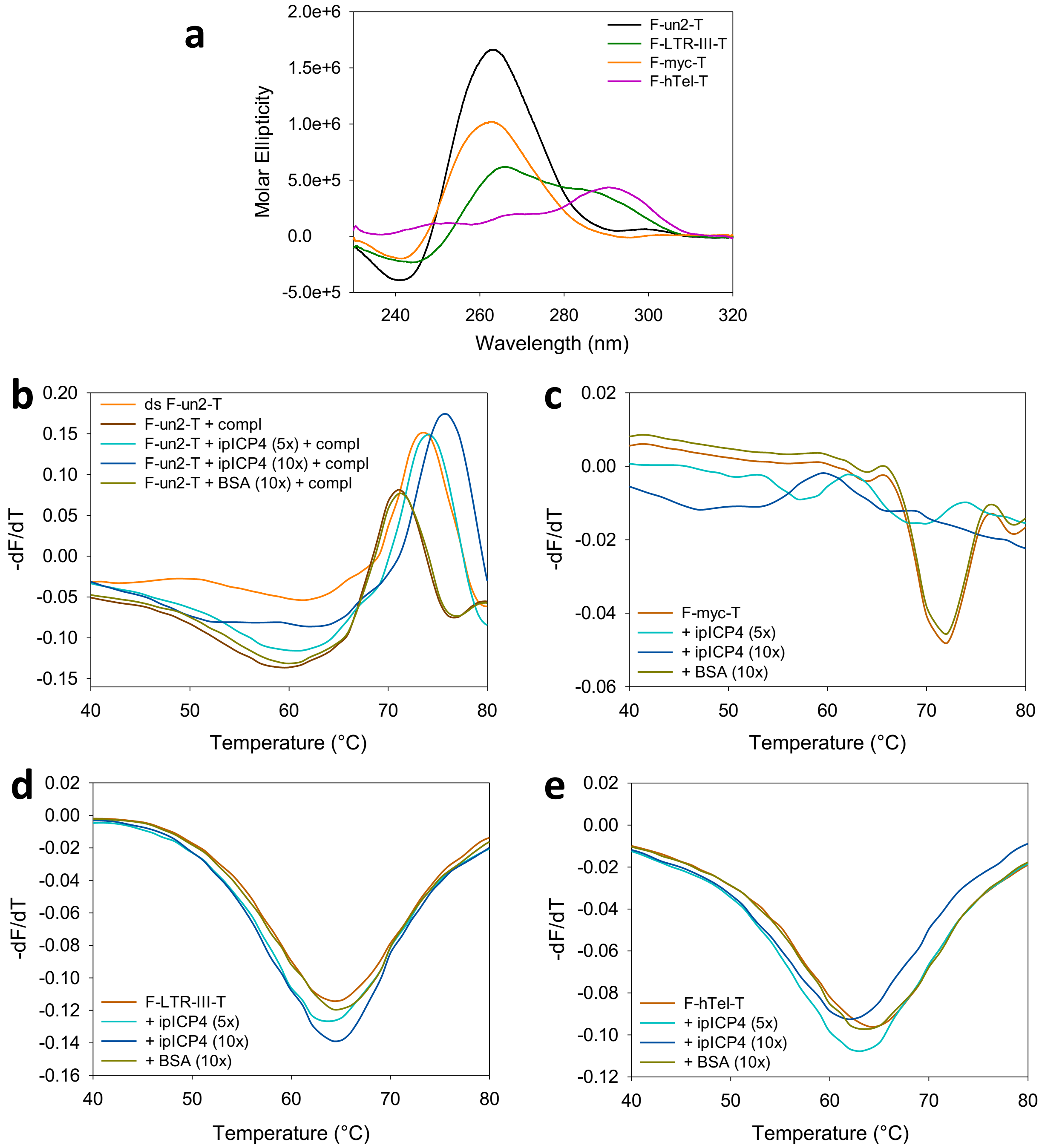
a
Molar Ellipticity
2.0e+6
1.5e+6
1.0e+6
5.0e+5
0.0
-5.0e+5
240
260
280
300
320
Wavelength (nm)
F-un2-T
F-LTR-III-T
F-myc-T
F-hTel-T
b
-dF/dT
Temperature (°C)
ds F-un2-T
F-un2-T + compl
F-un2-T + ipICP4 (5x) + compl
F-un2-T + ipICP4 (10x) + compl
F-un2-T + BSA (10x) + compl
c
-dF/dT
Temperature (°C)
F-myc-T
+ ipICP4 (5x)
+ ipICP4 (10x)
+ BSA (10x)
d
-dF/dT
Temperature (°C)
F-LTR-III-T
+ ipICP4 (5x)
+ ipICP4 (10x)
+ BSA (10x)
e
-dF/dT
Temperature (°C)
F-hTel-T
+ ipICP4 (5x)
+ ipICP4 (10x)
+ BSA (10x)

f

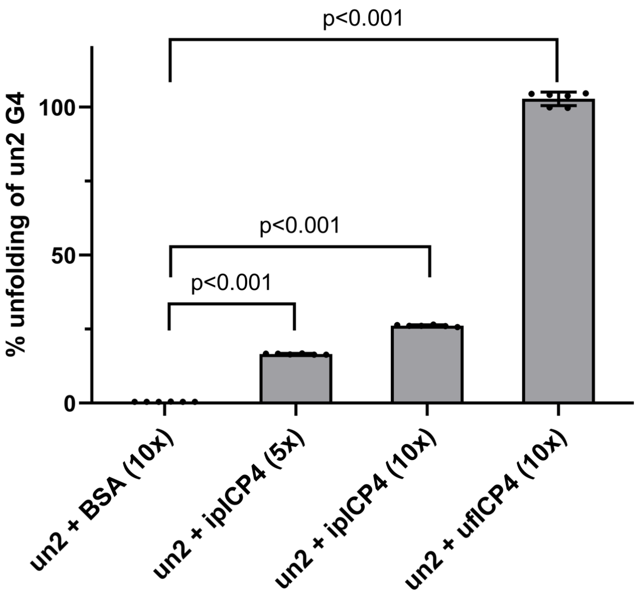
p<0.001
p<0.001
p<0.001
% unfolding of un2 G4
100
50
0
un2 + BSA (10x)
un2 + ipICP4 (5x)
un2 + ipICP4 (10x)
un2 + ufICP4 (10x)

g

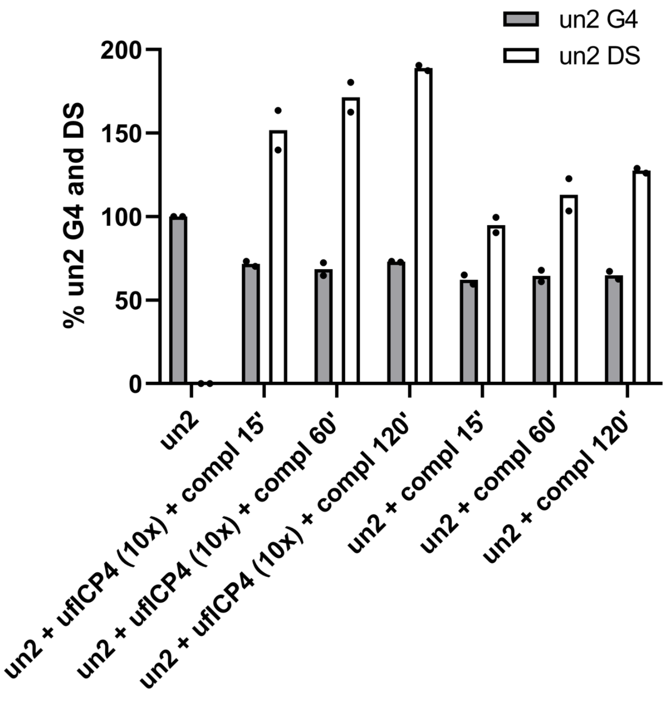
un2 G4
un2 DS
% un2 G4 and DS
200
150
100
50
0
un2
un2 + ufICP4 (10x) + compl 15'
un2 + ufICP4 (10x) + compl 60'
un2 + ufICP4 (10x) + compl 120'
un2 + compl 15'
un2 + compl 60'
un2 + compl 120'

h

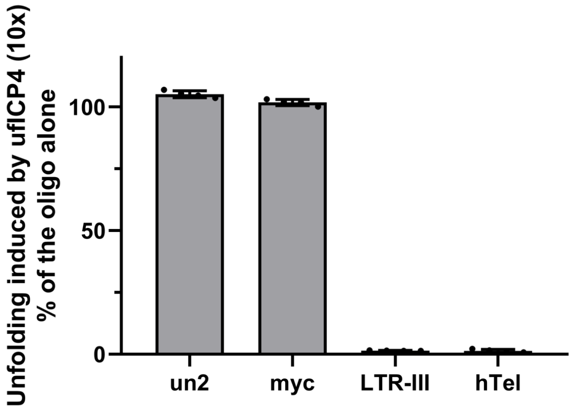

Figure S5. FRET melting. (a) CD spectra of the 5'-FAM/3'-TAMRA oligonucleotides used in the FRET melting experiments. The indicated labeled G4 oligonucleotides were folded in potassium phosphate buffer (un2 and myc 20 mM PB, 2 mM KCl; LTR-III and hTel 20mM PB, 80 mM KCl). Different KCl concentrations were used to reach the thermal denaturation of all tested oligonucleotides.). Oligonucleotide folding was tested in two independent assays, one replicate per condition. The figure shows spectra of one measurement per oligonucleotide. **(b-e)** FRET melting analysis of ICP4-mediated unfolding of G4-folded oligonucleotides. **(b)** First derivative FRET-melting curves (–dF_{525}/dT versus T) of the folded F-un2-T after incubation with both its complementary strand and ipICP4 (or BSA) at various protein/DNA ratios. The complementary strand and the protein were added at the same time to the G4-folded overnight and incubated for 30 min at 4°C before melting analysis. The complementary strand was used at 1:1 ratio with the G4-folded oligo. As control, un2 denatured and annealed to its complementary strand (1:1 ratio) overnight to yield the full ds oligonucleotide was used. **(c-e)** First derivative FRET-melting curves (–dF_{525}/dT versus T) of the indicated G4 oligonucleotides treated with ipICP4 or BSA as control at various protein/DNA ratios, in 20 mM PB pH 7.4 and 2-80 mM KCl. In **(b-e)** FRET melting analysis was performed in two independent experiments, with two replicates per condition. The figure shows one representative experiment per oligonucleotide. **(f)** Bar graph reporting the % of unfolding of un2 G4 incubated in the presence of ip/uf ICP4 or BSA at 4 °C for 45 min. Unfolding values were calculated based on the un2 radius values in the absence of protein. **(g)** Bar graph reporting the % of unfolding of un2 G4 incubated in the presence of ip/uf ICP4 or BSA and the complementary strand at 4 °C for 45 min. Unfolding values were calculated on the un2 Radius values in the absence of protein and complementary strand. Bar graphs **(f-g)** report the results of three independent experiments, with two replicates per condition. Mean values, P-values and SD are reported.

(h) Bar graph reporting the % of unfolding of all tested oligonucleotides incubated in the presence of ufICP4 (10x) for 30 min at 4 °C, and subsequently measured at 37°C (average of two experiments; bars represent standard deviation). The unfolding values are referred to the single tested oligonucleotides in the absence of protein. Oligonucleotide samples without protein were treated with the protein solvent (20 mM PB 100 mM KCl) to avoid bias. Bar graph **(h)** reports the results of two independent experiments, with two replicates per condition. Mean values and SD are reported.

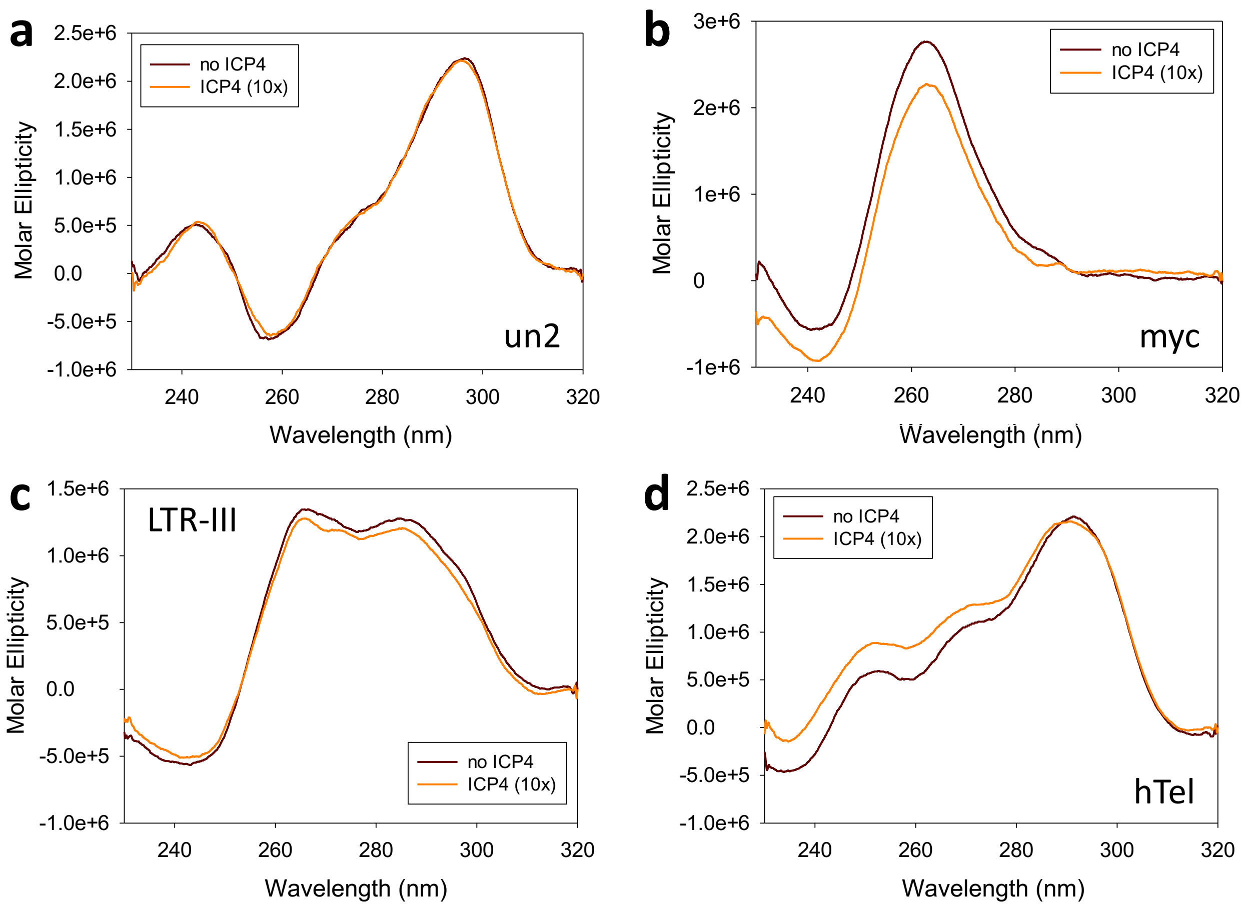

Figure S6. CD spectra in the presence/absence of ufICP4. The indicated unlabeled G4 oligonucleotides were folded in potassium phosphate buffer (un2 and myc 20 mM PB, 2 mM KCl; LTR-III and hTel 20 mM PB, 80 mM KCl) and incubated in the absence/presence of ufICP4 (10x protein/DNA ratio). Spectra of ICP4 alone was subtracted from G4-ICP4 complex spectra. Oligonucleotide folding was tested in two independent assays, one replicate per condition. The figure shows representative spectra per condition.

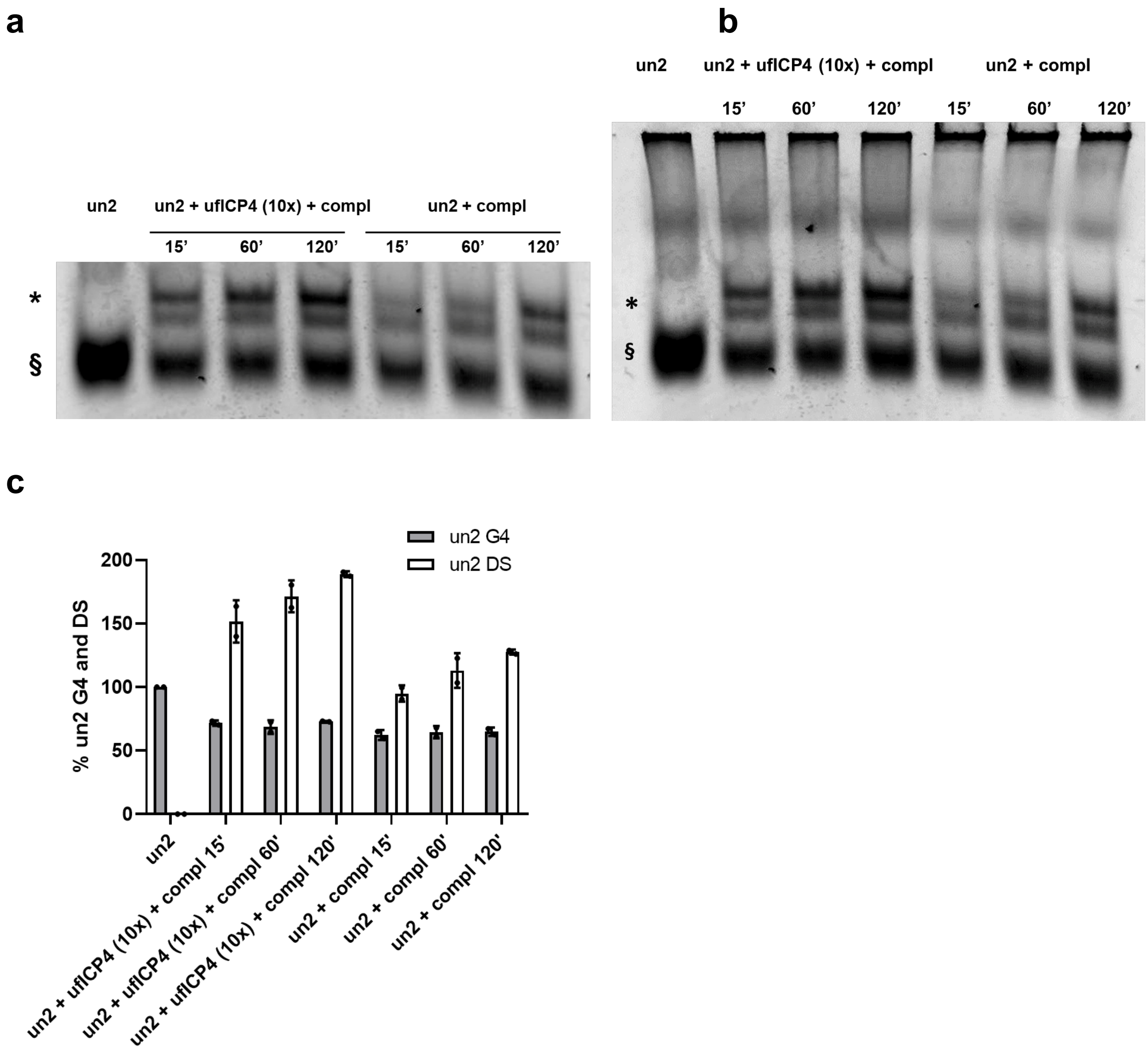

Figure S7: EMSA in the presence/absence of ufICP4 and complementary strand of un2 G4. **(a)** F-un2-T G4 was folded overnight in 20 mM PB and 2 mM KCl buffer and incubated with its complementary sequence (compl, 10-fold excess) in the presence or absence of ufICP4 (10x) for the indicated time (15-60-120 min). The native polyacrylamide gel was run in 50 mM PB buffer (pH 7.4) and the TAMRA signal was recorded at Thyphoon FLA 9500 (GE healthcare). G4-folded un2 (§) and duplex un2 (*) are indicated. **(b)** Uncropped EMSA native polyacrylamide gel showing F-un2-T G4 in presence and absence of ufICP4 and complementary strand). G4-folded un2 (§) and duplex un2 (*) are indicated. **(c)** Quantification of the G4 and the duplex bands are shown (average of two experiments; bars represent standard deviation).
EMSA and relative quantifications were performed in two independent experiments, one replicate per condition. The figure shows one representative EMSA, quantification shows mean values and SD.

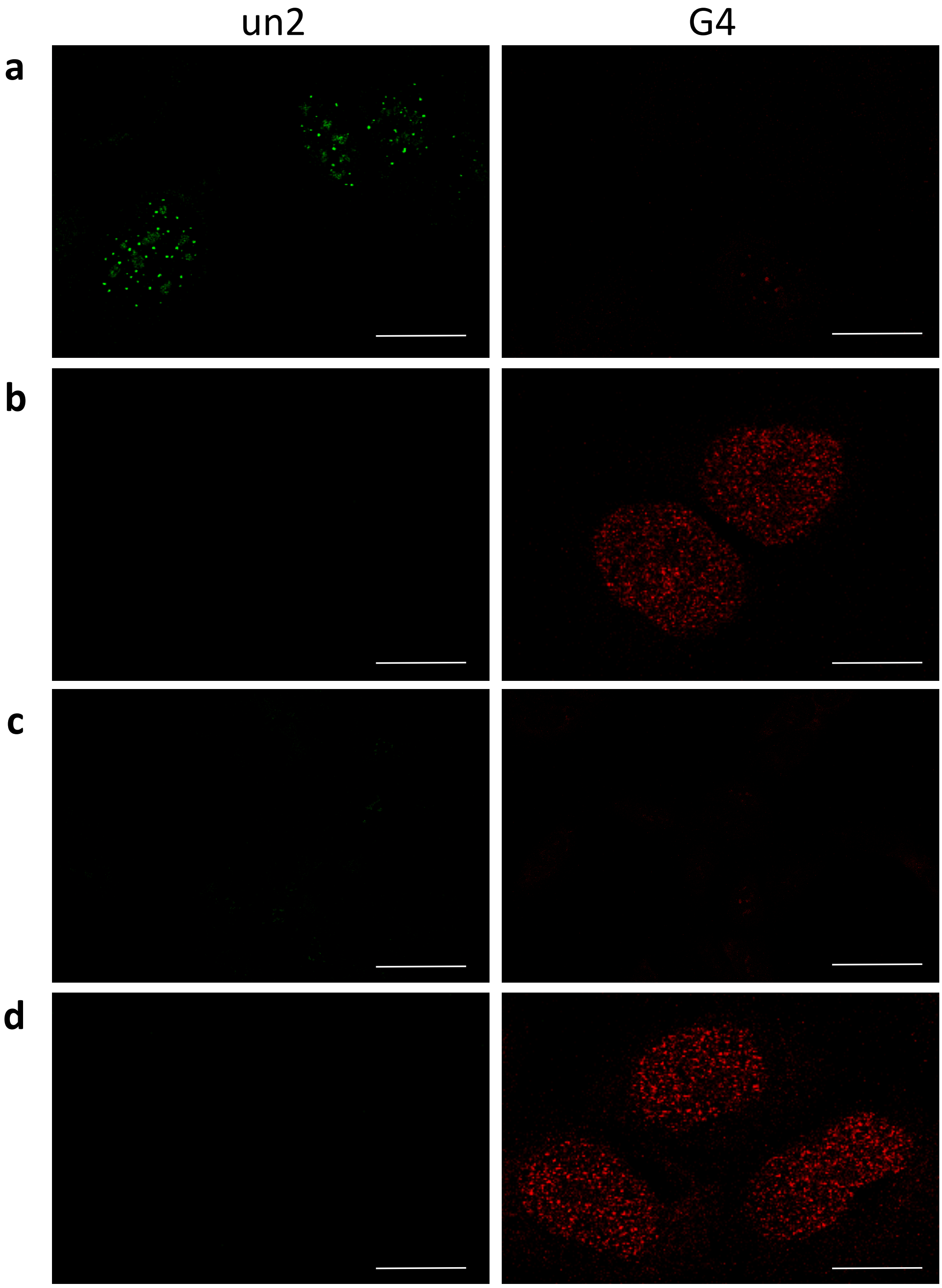

Figure S8. Controls of immune-FISH analysis. U-2 OS cells were infected with HSV-1, strain F (MOI 2,) and fixed at 8 hpi. **(a)** Control lacking 1H6 Ab for the detection of G4s; un2 probe and secondary Abs for the un2 probe and 1H6 Ab are present. **(b)** Control lacking the un2 probe; 1H6 Ab and secondary Abs for the un2 probe and 1H6 Ab are present. **(c)** Mock sample (non-infected U-2 OS cell) lacking 1H6 Ab; un2 probe and secondary Abs for the un2 probe and 1H6 Ab are present. **(d)** Mock sample (non-infected U-2 OS cell) lacking un2 probe; 1H6 Ab and secondary Abs for the un2 probe and 1H6 Ab are present. Immuno-DNA-FISH experiments were performed in three independent assays per tested condition. Scale bars 10 μm.

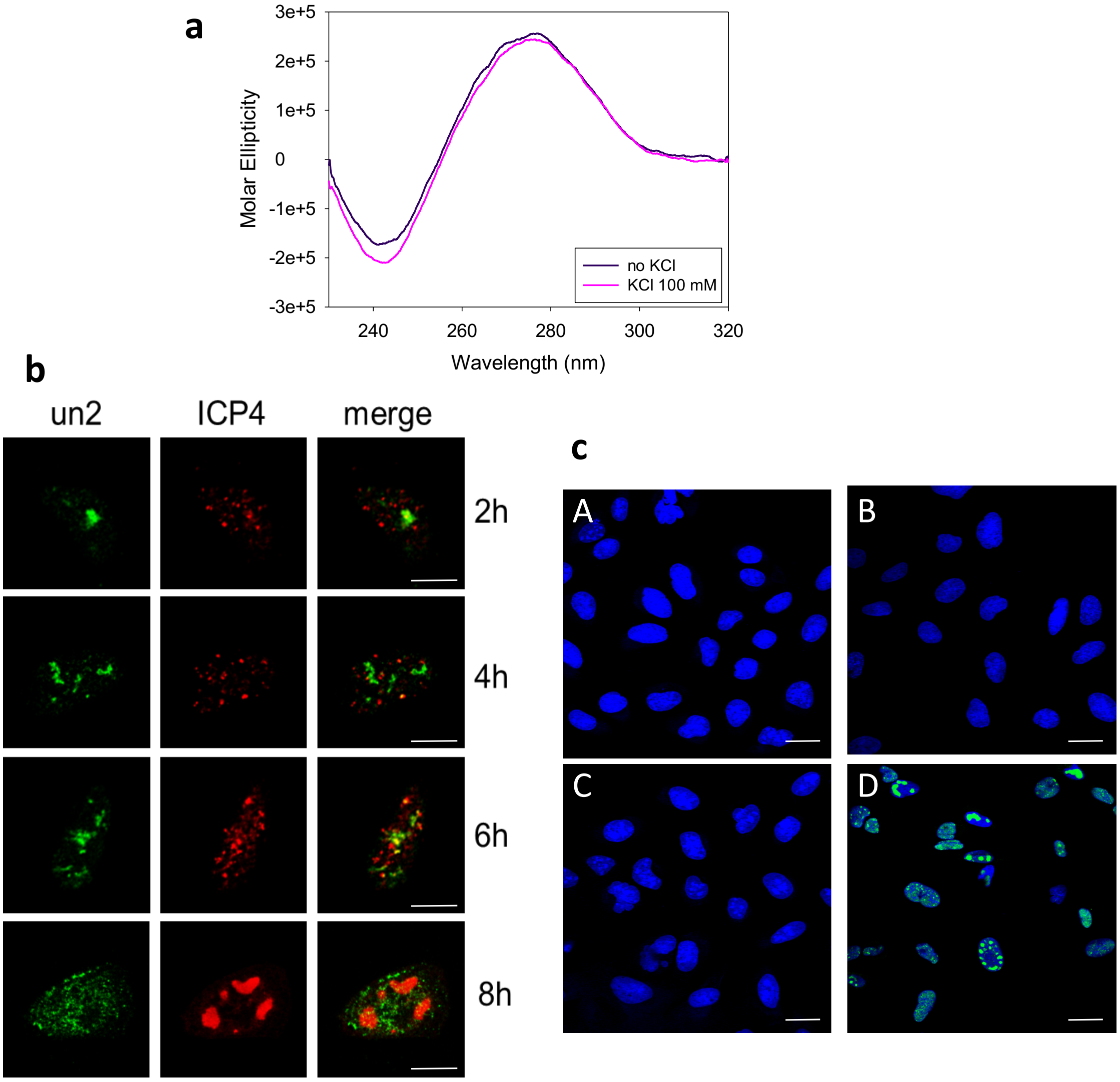

Figure S9. Interaction of ICP4 and un2 G4 in infected cells. **(a)** CD spectra of the un2 immuno-FISH and PLA probe that does not display a G4 signature. Oligonucleotide folding was tested in two independent assays, one replicate per condition. **(b)** Immunofluorescence images of ICP4 and un2 G4 colocalization in the nucleus of HSV-1 infected cells. Representative images of U2-OS cells were infected with HSV-1 strain F (MOI of 3) and assayed by immuno-DNA-FISH with the un2 probe (green) and the anti ICP4 antibody (red), fixed at 2-8 hpi. Merged signals are shown in yellow. Scale bars 10 μm. **(c)** PLA controls on mock (uninfected) U-2 OS cells (merged signals, blue staining detects nuclear nucleic acids): (A-C) mock cells incubated with un2 probe with (A) and without (C) primary antibodies and subjected to the PLA procedure; (B) mock cells incubated in the absence of un2 probe and with both primary and secondary antibodies and subjected to the PLA procedure. No false positive/unspecific PLA dots were observed; (D) U-2 OS infected cells (MOI = 3) processed for probe annealing (high temperature step) and incubated with the mouse anti-ICP4 primary antibody followed by the FITC-labeled secondary antibody (green signal) as control of the primary antibody ICP4 recognition in cells processed for PLA assay. Both Immuno-DNA-FISH and PLA experiments were performed in three independent assays per tested condition. Scale bars 20 μm.

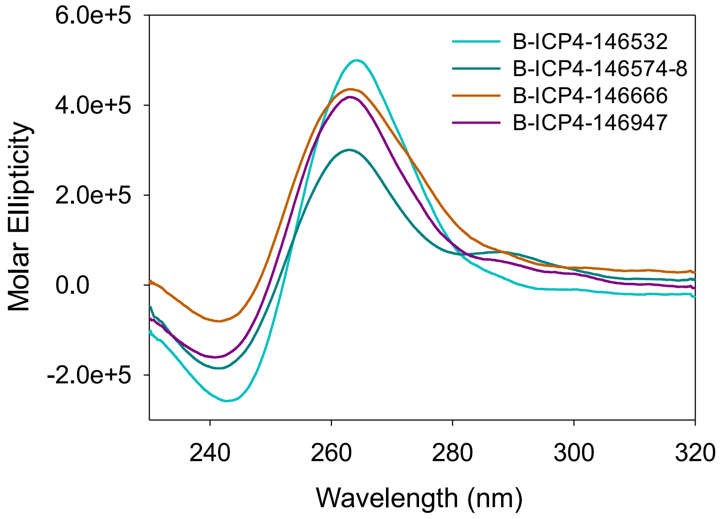

b

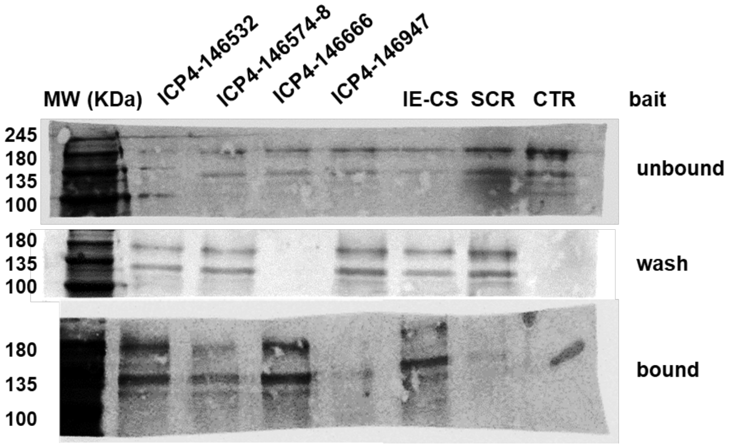

Figure S10. CD spectra of the biotinylated G4-forming oligonucleotides present in the ICP4 promoter and relative ICP4 binding affinity. (a) The oligonucleotides were folded in potassium phosphate buffer (20 mM PB, 80 mM KCl). Oligonucleotide folding was tested in two independent assays, one replicate per condition. The figure shows spectra of one measurement per oligonucleotide. **(b)** Uncropped Western blot analysis of ICP4 cross-linking pull-down towards the indicated four G4s embedded in the ICP4 promoter. IE-CS is the reported ICP4 consensus sequence [42,61,62]; SCR is a G-rich unfolded oligo; CTR is a control lane loaded with infected cell nuclear extracts not subjected to the pull-down procedure. The upper panel shows the unbound ICP4 fraction, the middle panel the washed out ICP4 fraction, the lower panel the ICP4 fraction bound to each oligo. The multiband appearance of ICP4 from HSV-1 infected cells in WB has been reported [63] and reflects the presence of differently post-transcriptionally processed isoforms. The experiment was independently performed in duplicate. Each replicate was tested once in each experiment.

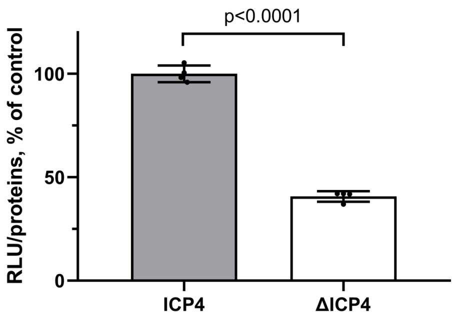

Figure S11. Luciferase activity of full-length and delated ICP4 promoter Luciferase assay of the full length and delated (nts 146629-146778, ΔICP4) promoter, lacking the ICP4 146666 G4 sequence in U2-OS cells transfected 24 h post seeding. Luciferase signal was measured 24 h post transfection. Results were collected from two independent experiments with two replicates per condition. Mean values, P-value and SD are reported.

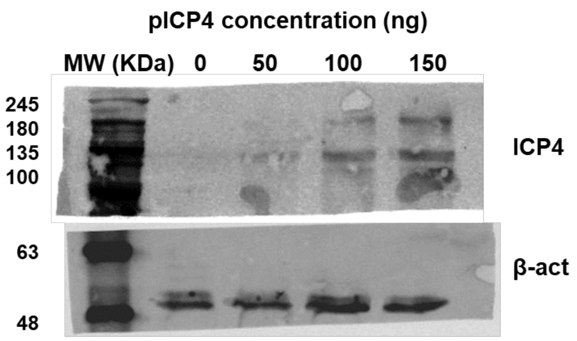

Figure S12. ICP4 expression in U-2 OS cells via plasmid transfection Uncropped Western blot analysis of ICP4 expression in the presence of increasing amounts of ICP4 expression plasmid in transfected U-2 OS cells. ICP4 and TK promoters were cloned in the promoterless pGL-4.10 plasmid upstream of the luciferase gene and transfected into U-2 OS cells. **β**-actin was used as housekeeping control for cell amount. Two independent experiments were performed with one replicate per condition in each experiment.

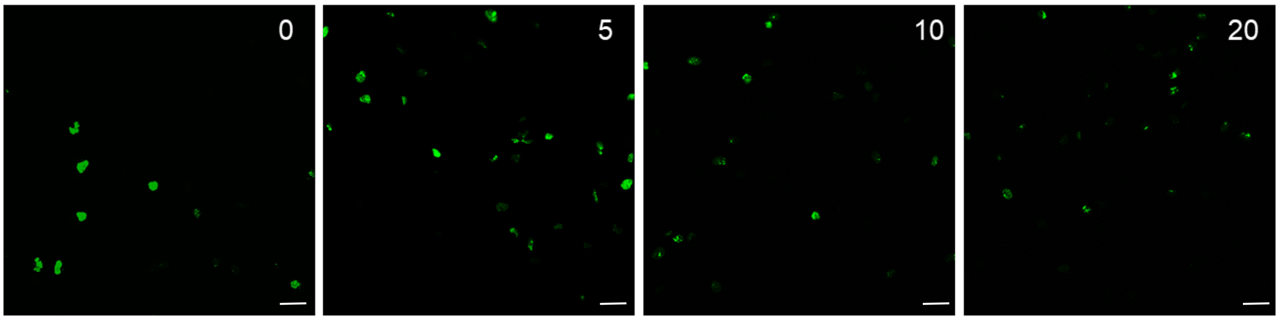

Figure S13. B19 effect on ICP4-YFP. Representative confocal microscopy images of U-2 OS cells transfected with ICP4-EYFP plasmid that express EYFP under the ICP4 promoter, in the presence of increasing concentrations of B19 (0, 5, 10, 20 μM, as indicated), at 40X magnification. The assay was performed in two independent experiments, one replicate per condition. Scale bars 20 μm.

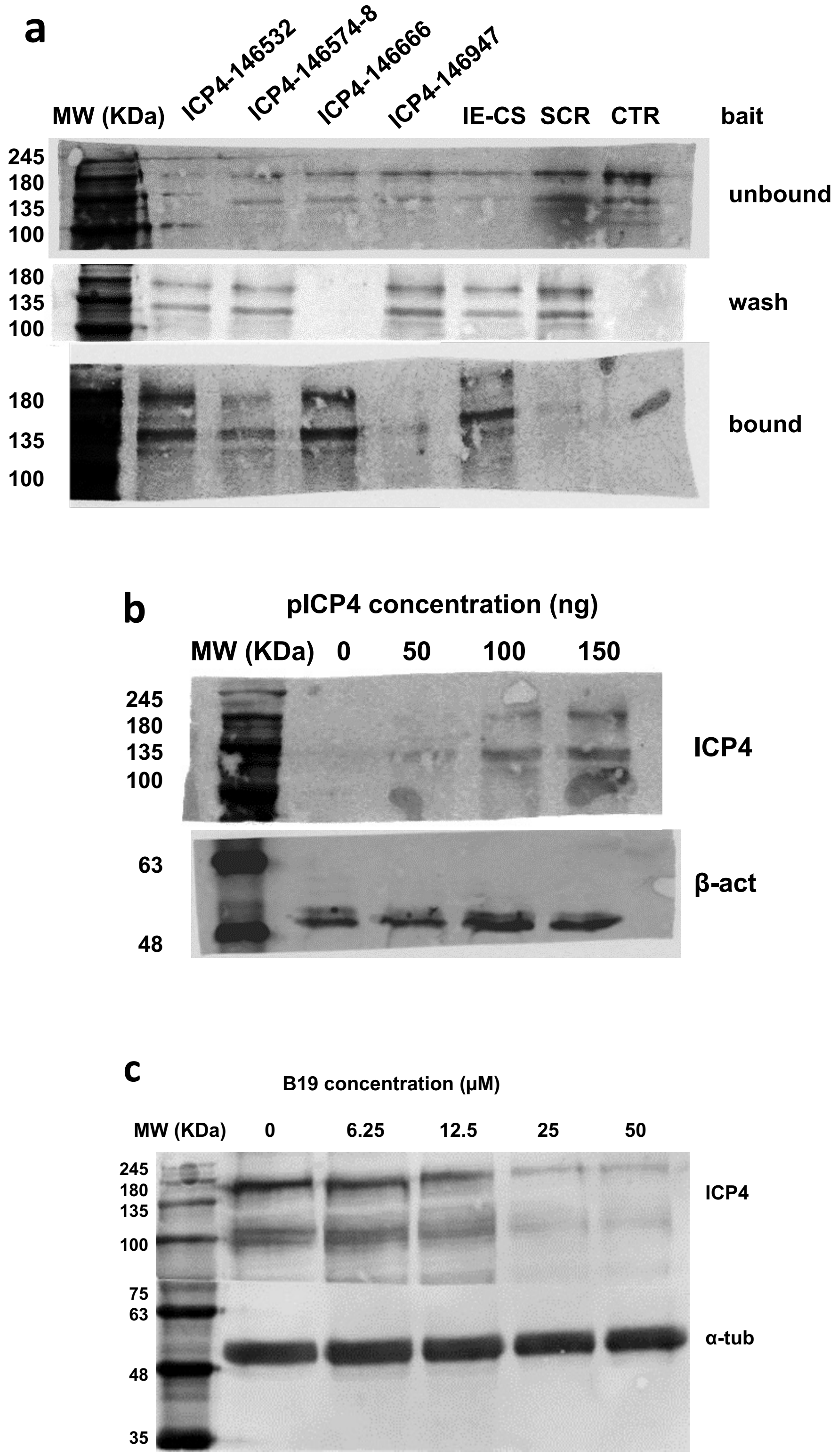

Figure S14. Uncropped original images of western blot gels in figures in the main text. **(a)** Figure 3b in the main text. **(b)** Figure 4a in the main text. **(c)** Figure 5d in the main text.
