# Supplementary material for: Parallel G-quadruplexes recruit the HSV-1 transcription factor ICP4 to promote viral transcription in herpes virus-infected human cells
Source: Commun Biol. 2021 Apr 30;4:510. doi: 10.1038/s42003-021-02035-y (PMC8087788; doi:10.1038/s42003-021-02035-y)
Supplement: Supplementary file 2 — Description of Additional Supplementary Files [file 42003_2021_2035_MOESM2_ESM.pdf]

Description of additional supplementary files

File name: Supplementary Data 1

Description: Source data excel file for all graphs and figures present in the main manuscript and supporting information.
