# Supplementary material for: Parallel G-quadruplexes recruit the HSV-1 transcription factor ICP4 to promote viral transcription in herpes virus-infected human cells
Source: Commun Biol. 2021 Apr 30;4:510. doi: 10.1038/s42003-021-02035-y (PMC8087788; doi:10.1038/s42003-021-02035-y)
Supplement: Supplementary file 4 — Reporting Summary [file 42003_2021_2035_MOESM4_ESM.pdf]

nature research

Corresponding author(s): Sara N. Richter

Last updated by author(s): March 18, 2021

Reporting Summary

Nature Research wishes to improve the reproducibility of the work that we publish. This form provides structure for consistency and transparency in reporting. For further information on Nature Research policies, see our Editorial Policies and the Editorial Policy Checklist.

Statistics

For all statistical analyses, confirm that the following items are present in the figure legend, table legend, main text, or Methods section.

n/a	Confirmed	
☐	☒	The exact sample size (n) for each experimental group/condition, given as a discrete number and unit of measurement
☐	☒	A statement on whether measurements were taken from distinct samples or whether the same sample was measured repeatedly
☐	☒	The statistical test(s) used AND whether they are one- or two-sided<br>*Only common tests should be described solely by name; describe more complex techniques in the Methods section.*
☒	☐	A description of all covariates tested
☒	☐	A description of any assumptions or corrections, such as tests of normality and adjustment for multiple comparisons
☒	☐	A full description of the statistical parameters including central tendency (e.g. means) or other basic estimates (e.g. regression coefficient) AND variation (e.g. standard deviation) or associated estimates of uncertainty (e.g. confidence intervals)
☒	☐	For null hypothesis testing, the test statistic (e.g. F, t, r) with confidence intervals, effect sizes, degrees of freedom and P value noted<br>*Give P values as exact values whenever suitable.*
☒	☐	For Bayesian analysis, information on the choice of priors and Markov chain Monte Carlo settings
☒	☐	For hierarchical and complex designs, identification of the appropriate level for tests and full reporting of outcomes
☒	☐	Estimates of effect sizes (e.g. Cohen's d, Pearson's r), indicating how they were calculated

Our web collection on statistics for biologists contains articles on many of the points above.

Software and code

Policy information about availability of computer code

Data collection	No software was used
Data analysis	Sigma Plot Version 12.3, ImageJ Version 1.52, Graph Prism 8

For manuscripts utilizing custom algorithms or software that are central to the research but not yet described in published literature, software must be made available to editors and reviewers. We strongly encourage code deposition in a community repository (e.g. GitHub). See the Nature Research guidelines for submitting code & software for further information.

Data

Policy information about availability of data

All manuscripts must include a data availability statement. This statement should provide the following information, where applicable:

- Accession codes, unique identifiers, or web links for publicly available datasets
- A list of figures that have associated raw data
- A description of any restrictions on data availability

The authors declare that [the/all other] data supporting the findings of this study are available within the paper [and its supplementary information files].

Field-specific reporting

Please select the one below that is the best fit for your research. If you are not sure, read the appropriate sections before making your selection.

☒ Life sciences ☐ Behavioural & social sciences ☐ Ecological, evolutionary & environmental sciences

For a reference copy of the document with all sections, see nature.com/documents/nr-reporting-summary-flat.pdf

Life sciences study design

All studies must disclose on these points even when the disclosure is negative.

Sample size	Protein binding experiments and colocalization studies were performed in biological duplicates or triplicates as stated in the text. The consistently very high correlation between these replicates, suggests that this is sufficient.
Data exclusions	No data were excluded
Replication	Protein binding and colocalization experiments were highly reproducible, as stated above. All circular dichroism, FRET, gene reporter, drug treatment studies were successfully replicated at least two times in independent experiments, and in each experiment three repetitions for each sample were obtained. For colocalization, circular dichroism and FRET analysis, one representative experiment is displayed. For reporter gene expression and antiviral assays standard deviation is reported.
Randomization	Experimental replicates were performed on different days.
Blinding	Experimental replicates or some steps within experiments composed of several steps were sometimes performed by different people.

Reporting for specific materials, systems and methods

We require information from authors about some types of materials, experimental systems and methods used in many studies. Here, indicate whether each material, system or method listed is relevant to your study. If you are not sure if a list item applies to your research, read the appropriate section before selecting a response.

Materials & experimental systems

n/a	Involved in the study	
☐	☒	Antibodies
☐	☒	Eukaryotic cell lines
☒	☐	Palaeontology and archaeology
☒	☐	Animals and other organisms
☒	☐	Human research participants
☒	☐	Clinical data
☒	☐	Dual use research of concern

Methods

n/a	Involved in the study	
☒	☐	ChIP-seq
☒	☐	Flow cytometry
☒	☐	MRI-based neuroimaging

Antibodies

Antibodies used	anti-G4 antibody 1H6, gift from the producer; anti-ICP4mouse monoclonal H943, Santa Cruz, Biotechnology; anti-biotin antibody Bethyl Laboratories; secondary antibodies anti-mouse Alexa 488 and anti-rabbit Alexa 546, ThermoFisher Scientific.
Validation	All antibodies were preliminary tested for activity with appropriate controls: controls have been inserted in the experiment and images.

Eukaryotic cell lines

Policy information about cell lines

Cell line source(s)	Human bone osteosarcoma cells (U-2 OS) from the European Collection of Authenticated Cell Cultures (ECACC) were purchased from Sigma Aldrich, Milan, Italy.
Authentication	The cell line was not authenticated in the lab, however it was purchased from a database of authenticated cell cultures and used within few passages.
Mycoplasma contamination	Cells are routinely tested for mycoplasm contamination on a monthly basis.
Commonly misidentified lines (See ICLAC register)	None
